# Cicatricial pemphygoid in the upper aerodigestive tract: report of two cases

**DOI:** 10.1016/S1808-8694(15)30158-0

**Published:** 2015-10-18

**Authors:** Vinicius Antunes Freitas, Helena Maria Gonçalves Becker, Roberto Eustáquio Santos Guimarães, Letícia Paiva Franco, Fernando Fernandes Guimarães, Marcelo Figueredo Martins, Paulo Fernando Tormin Crosara

**Affiliations:** a MD, specialization student on Otorhinolaryngology at Núcleo de Otorrino BH; b PhD, Adjunct Professor in the Department of Otorhinolaryngology, Ophthalmology, and Speech and Hearing Therapy at the UFMG Medical School; c PhD, Adjunct Professor in the Department of Otorhinolaryngology, Ophthalmology, and Speech and Hearing Therapy at the UFMG Medical School; d Specialist on Otorhinolaryngology, ENT at Hospital das Clínicas da UFMG; e Specialist on Otorhinolaryngology, Otorhinolaryngologist; f MD, Resident on General Practice; g PhD, Substitute Professor in the Department of Otorhinolaryngology, Ophthalmology, and Speech and Hearing Therapy at the UFMG Medical School. ENT Service of the Hospital das Clínicas da Universidade Federal de Minas Gerais

**Keywords:** scars, pemphygoid, aerodigestive tract

## Abstract

Cicatricial pemphygoid (mucous membrane cicatricial pemphygoid) is a chronic autoimmune inflammatory disease characterized by subepithelial bubbles in mucous membranes and, occasionally on the skin. It may affect the mouth, the nose, pharynx, larynx, the eyes, esophagus, anus, genitals and skin; especially affecting patients between fifty and sixty years of life. Treatment includes systemic steroids and immunosuppressive agents. In the present paper we describe two cases with the active disease, and one of them had sepsis because of using immunosuppressive agents and another that presented supraglottic stenosis requiring tracheostomy.

## INTRODUCTION

Cicatricial pemphigoid, also known as mucous membrane cicatricial pemphigoid, is a rare autoimmune disease of chronic development that involves mainly mucous membranes with the formation of subepithelial blisters. Subjects in older age groups (mean 70 years) are predominantly affected, although cases involving younger patients have been reported. This disease is uncommon among non-Caucasians, and is more frequently found in men^4^, ^5^, ^6^.

Diagnosis requires observation of typical clinical manifestations and pathology tests indicating the presence of linear immune deposits of IgG, IgA, and/or C3 on the epithelial basal membrane^1^.

Few publications in the literature describe this disease from the standpoint of otorhinolaryngology. Most papers report on ophthalmic and dermatologic cases. The most frequent therapies are cyclophosphamide combined with prednisone for high risk patients (defined here in a simplified manner as presence of ocular, genital, nasopharyngeal, esophageal, or laryngeal involvement) and dapsone for less severe cases^1^, ^4^. Azathioprine was also considered as an option for high risk cases in spite of reports mentioning disease exacerbation consequent to its use 3. Subconjunctival mitomycin-C has also been described in cicatricial pemphigoid eye lesions^5^.

Cicatricial pemphigoid combined with laryngeal involvement must be initially treated clinically, as surgery should be reserved for more severe cases with airway obstruction due to frequent disease recurrence^6^.

## CASE REPORT

### Case 1

J.R.S., 52, brown, driver. The patient started having repetition epistaxis six years ago, along with nasal obstruction and weight loss. The patient lost 18 kilograms in one year. He claimed not to have had other diseases previously. He had granulous nasal septum and synechia obstructing 80% of the left nasal fossa, apart from granulation in the soft palate. Biopsy was done on septal mucosa and palate tissue. Alcohol-acid resistant bacteria tests and chest x-rays were normal. Pathology tests indicated the presence of amastigote Leishmania sp. The patient was sent to the department of infectious diseases and was treated with two cycles of Glucantime and then amphotericin B, with no improvement. Bullous lesions and ulcers on the left elbow and lower limbs began to appear, and the Dermatology Department indicated the patient could have cicatricial pemphigoid combined with cutaneous leishmaniasis.

About a year after the first visit, the patient came back with jugal mucosa lesions, and pemphigus vulgaris was also considered. The patient had been followed by the ophthalmology service to treat a non-specific chronic conjunctivitis, and cicatricial pemphigoid was once again considered.

Treatment with steroids was started as ocular and oral lesions persisted.

The following month oral lesions were found by the dermatology service, and azathioprine combined with prednisone was administered. Two months later the oral lesions were gone, but the patient had active eye disease despite the increase on the dosage of immunosuppressive medication kept for about ten months. The patient stopped taking azathioprine and started with cyclophosphamide.

Five months after he began taking the new medication, the patient developed severe complications as a result of chronic immunosuppressant and steroid therapy (cerebellar abscess, meningitis, lymphadenopathy with effusion, herpes zoster, and costal arch, humerus, and lumbar vertebrae pathologic fracture) and had to be hospitalized for about three months. During hospitalization cyclophosphamide was stopped and prednisone doses were decreased. The patient was discharged as the lesions disappeared, with however reduced visual acuity.

In the last two years he had cataract in the left eye and nasal synechia. Tests (nasal fibroscopy and computerized tomography) showed synechia in the left nasal fossa and no granulation in the mucosa. The patient is currently not taking any medication and is in otorhinolaryngological review. In March of 2004 he had no signs of active disease.

### Case 2

T.M.S., 78, Caucasian. The patient began experiencing painful, bullous lesions in the oral cavity thirteen years ago, compromising food intake and leading the patient to lose 21 kilograms in three months. The patient went to a series of specialized care providers but remained undiagnosed. She had been operated previously for glaucoma and cataract. Months after the onset of the oral lesions she had ophthalmic complications and was suspected for conjunctival inflammation secondary to a foreign body (surgical suture). In a subsequent visit the patient had a more exuberant disease and was suspected for cicatricial pemphigoid.

About 18 months after the lesions appeared, she was being followed at the dermatology service and started taking thalidomide only to partially improve. Next, prednisone was added. She was in remission for another two months and then stopped taking thalidomide. Back then her diagnosis was undefined, and in a visit with an ophthalmologist cicatricial pemphigoid was once again considered. An ENT assessment done in August of 1993 showed bullous lesions in the oral cavity and granulation in the left nasal fossa.

In 1996, five years after symptom onset, the patient”s ophthalmic complications were in remission and she had no high digestive tract lesions.

**Figure 1:**
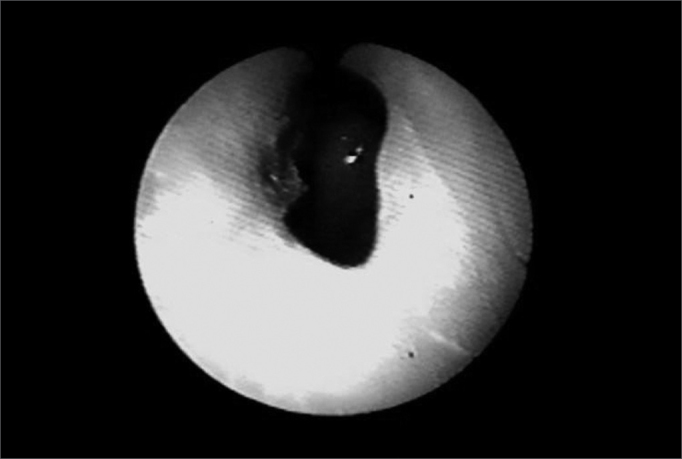
Nasal cavity stenosis

**Figure 2:**
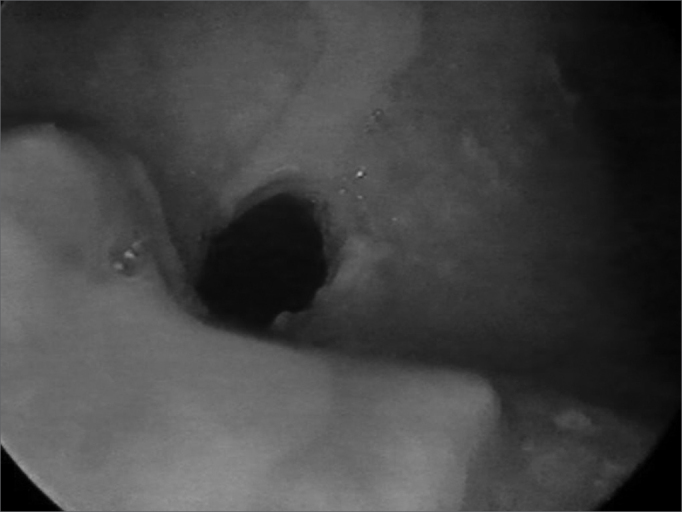
Supraglottal stenosis

In 1999 she started taking an immunosuppressant (azathioprine) and reduced her steroid dosage. In 2003, dapsone was added to azathioprine and prednisone. The patient then started having frequent dyspnea episodes. She was sent to the pneumology service and spirometry tests indicated she had a high respiratory obstruction. She was then sent to the ENT service, where stenosis in the left nostril was found (Fig. 1), nasal crust to the right and bullous/vesicular lesions in the nasal septum mucosa; soft palate blisters/vesicles and cicatricial lesions in the soft and hard palate. Indirect laryngoscopy showed a thickened epiglottis; her vocal chords could not be visualized. Nasal fibroscopy showed extensive membrane-shaped supraglottal stenosis with an orifice of about 5 millimeters close to the glottis (Fig. 2). The glottis was not involved. Tracheostomy was performed and her dyspnea improved. Six months elapsed between the onset of the dyspnea episodes and the tracheostomy. Today, in August of 2004, the patient is tracheostomized and without oral cavity lesions. She is taking azathioprine, prednisone, and dapsone.

## DISCUSSION

This is a rare condition that involves the oral cavity (100%), followed by the eyes (61-80%), pharynx (43%), nose (38%), larynx (30%), genitalia (20-30%), rectum (11%), esophagus (7%) and sometimes the skin (10-43%)^10^. Eye lesions may evolve to blindness, while upper airway involvement may lead to obstruction. Steroid therapy, when employed, may also introduce eye disorders. Our patients developed the most frequently found lesions, and had no skin, genitalia, or digestive tract involvement. As seen in both cases, diagnosis can be tricky and differentiation is required from a number of conditions, thus requiring a multidisciplinary approach. Both cases required a number of biopsies before a definitive diagnosis could be produced (clinical history and immunohistopathology tests). Biopsies were done using a cup forceps. Clinical treatment is paramount in preventing recurrence. However, patients are mostly older and susceptible to drug adverse effects. The first patient was treated with cyclophosphamide and prednisone after taking other immunosuppressant drugs and was in remission, despite some severe complications. The patient was in complete remission, i.e., with improved quality-of-life, no longer hospitalized, and had synechia in the left nasal fossa. She had no need for surgery due to the residual post-remission manifestations (‘scars”), as they were quite modest. She had left eye cataract, thus stressing the need for multidisciplinary complication care. The second case was and elderly Caucasian female. She was initially treated with thalidomide and prednisone, and is now taking azathioprine, prednisone, and dapsone. Her disease is still active and she is tracheostomized due to obstructive complications. Cyclophosphamide combined with prednisone is the first choice therapy to reduce supraglottal stenosis and promote disease remission (high risk cases), although the patient was clinically stable given the tracheostomy, which at first is there to resolve her respiratory problems. Mitomycin-C could have been used as an alternative to treat active eye lesions, and would possibly be effective against airway stenosis. Inducing disease remission is fundamental, thus avoiding the need for surgical intervention as well as periodic assessment to prevent recurrence, and disease and treatment complications.

## CONCLUSION

Mucous membrane cicatricial pemphigoid must be considered when bullous chronic or cicatricial lesions involve the oral mucosa at first, followed by the pharyngeal, nasal, and laryngeal mucosa, mainly of elderly patients. ENT examination and nasal fibroscopy to periodically assess lesions (activity/remission) and detect complications (stenosis, synechia) are required. Early diagnosis and treatment are useful in preventing complications.
